# Peripheral CD8+ T Cell Dynamics and Clinical Outcomes in Metastatic Non-Small Cell Lung Cancer Following Bronchoscopic Cryotherapy and Pembrolizumab-Based Therapy

**DOI:** 10.3390/cancers18111793

**Published:** 2026-05-31

**Authors:** Gediminas Vasiliauskas, Evelina Žemaitė, Erika Skrodenienė, Lina Poškienė, Skaidrius Miliauskas, Marius Žemaitis

**Affiliations:** 1 Department of Pulmonology, Lithuanian University of Health Sciences, 44307 Kaunas, Lithuania; 2 Department of Laboratory Medicine, Lithuanian University of Health Sciences, 44307 Kaunas, Lithuania; 3 Department of Pathology, Lithuanian University of Health Sciences, 44307 Kaunas, Lithuania

**Keywords:** non-small cell lung cancer, cryotherapy, immunotherapy, flow cytometry, CD8+ T cells

## Abstract

**Background**: Bronchoscopic cryotherapy may enhance anti-tumor immunity and improve the effect of immune checkpoint blockade, but CD8+ T cell dynamics after cryotherapy combined with pembrolizumab-based therapy in metastatic non-small cell lung cancer (NSCLC) remain insufficiently characterized. **Methods**: In this prospective, exploratory, randomized, controlled, single-center study, metastatic NSCLC patients were assigned to bronchoscopic cryotherapy performed 7 ± 1 days before first-line pembrolizumab-based therapy or to standard treatment alone. Peripheral blood mononuclear cells were analyzed by flow cytometry at baseline, week 3, and week 6. CD8+ T cell subsets defined by CD45RO, CD28, granzyme B (GzB), IFNγ, Ki-67, and PD-1 were evaluated in relation to treatment group, radiologic response, progression-free survival (PFS), and overall survival (OS). **Results**: Flow cytometry was performed in 76 patients, including 34 in the cryotherapy group and 42 in the control group. Cryotherapy was associated with a treatment-specific increase in circulating GzB+ CD8+ T cells by week 6. In contrast, Ki-67+ CD8+ and GzB+Ki-67+ cells increased in both treatment groups, suggesting that early peripheral CD8+ proliferation was largely shared across pembrolizumab-based therapy. Radiologic responders demonstrated more sustained proliferative CD8+ dynamics, most consistently reflected by increased CD28+ Ki-67+ cells. In exploratory landmark survival analyses, a higher week-3-to-baseline GzB+ Ki-67+ CD8+ ratio showed a hypothesis-generating association with longer PFS and OS. **Conclusions**: In metastatic NSCLC patients receiving first-line pembrolizumab-based therapy, the addition of bronchoscopic cryotherapy was associated with peripheral CD8+ T cell remodeling toward enhanced cytotoxic activity. Proliferative CD8+ T cell changes were associated with radiologic response and better survival. These findings support bronchoscopic cryotherapy as a potential immune-modulating adjunct and warrant validation in larger studies.

## Simple Summary

Bronchoscopic cryotherapy is routinely used to destroy tumor tissue in the airways, but it may also help the immune system recognize and fight cancer more effectively. In this study, we examined blood samples from patients with metastatic non-small cell lung cancer who received cryotherapy before pembrolizumab-based treatment and compared them with samples from patients who received standard treatment alone. We focused on CD8-positive T cells, which play an important role in killing cancer cells. We found that cryotherapy was linked to signs of stronger anti-tumor immune activity, while early increases in dividing CD8-positive T cells were associated with better treatment outcomes. These findings may help guide future research on combining local tumor treatment with immunotherapy and on developing blood-based markers of treatment benefit.

## 1. Introduction

Lung cancer continues to significantly affect the global cancer burden, as the most commonly diagnosed malignancy and the leading cause of cancer death worldwide [1]. Non-small cell lung cancer (NSCLC) comprises the vast majority of lung cancers and is still frequently diagnosed at an advanced stage, where durable disease control is difficult to achieve [2,3].

The introduction of Programmed cell death protein 1/Programmed death-ligand 1 (PD-1/PD-L1) checkpoint blockade has transformed the treatment landscape for advanced NSCLC, with various immunotherapy-based regimens now essential for the treatment of multiple lung cancer settings [4]. Even so, durable benefit is confined to a subset of patients, and both primary resistance and later progression remain common [5,6]. This highlights the need for combination strategies that can enhance immune priming, as well as the lack of dynamic biomarkers that capture successful immune anti-tumor activation more effectively than static baseline variables.

One possible method to intensify immune priming is to combine checkpoint inhibition with local tumor destruction. Bronchoscopic cryotherapy is already used in interventional pulmonology for airway recanalization and endobronchial tumor debulking, but its role may extend beyond mechanical cytoreduction [7,8,9]. Recent translational and clinical work has reframed cryotherapy as a potential in situ immune-conditioning approach with cryogenic tumor injury, releasing intact tumor neoantigens and promoting a more favorable immune environment for checkpoint blockade [8,9,10,11].

In this context, CD8+ T cells are especially relevant for their central role in effective anti-tumor immunity. The state of these cells, such as activation, proliferation, and exhaustion states, as well as cytotoxic activity, all shape the likelihood of response to immunotherapy [12,13]. Taking these considerations into account, we postulated that the interaction between cryotherapy and immunotherapy may benefit clinical outcomes in patients with metastatic NSCLC and could be best understood by focusing on CD8+ T cells.

## 2. Materials and Methods

### 2.1. Study Design, Setting, and Treatment Procedures

This translational analysis was conducted within a prospective, randomized, controlled, single-center study at the Hospital of the Lithuanian University of Health Sciences, Kauno klinikos, Kaunas, Lithuania. Inclusion and exclusion criteria are described in an earlier publication [14]. In summary, adult patients with histologically confirmed metastatic NSCLC, known tumor PD-L1 status, no activating Epidermal growth factor receptor or Anaplastic lymphoma kinase alterations, and an Eastern Cooperative Oncology Group (ECOG) performance status of 0–1 were screened for inclusion. To be eligible, patients also had to have at least one pulmonary lesion technically reachable by flexible bronchoscopy and be candidates for first-line pembrolizumab-based systemic treatment. Patients were excluded if they were unable to undergo bronchoscopy, had prior immunotherapy, had received recent anticancer treatment, or had active autoimmune, infectious, or immunosuppressive conditions that could interfere with protocol therapy or immune monitoring.

The trial was approved by the Kaunas Regional Biomedical Research Ethics Committee (Protocol No. BE-2-14, 18 January 2023), conducted in accordance with the Declaration of Helsinki, and registered at ClinicalTrials.gov (NCT06000358). All participants provided written informed consent before enrollment.

Participants were assigned in a 1:1 ratio to bronchoscopic cryotherapy followed by standard first-line pembrolizumab-based treatment or to standard treatment alone. The present report focuses on peripheral blood CD8+ T cell immunophenotyping.

Patients allocated to the cryotherapy arm underwent bronchoscopic cryotherapy 7 ± 1 days prior to the initiation of systemic treatment. Cryotherapy (Erbe Elektromedizin GmbH, Tübingen, Germany) was delivered through a flexible bronchoscope (Olympus Corporation, Tokyo, Japan) under direct endobronchial visualization for central lesions or under radial endobronchial ultrasound and fluoroscopic guidance for transbronchial access to peripheral lesions. Carbon dioxide was used as the cryogenic agent. Each application consisted of a 60 s freezing phase followed by passive thawing for 60 s, repeated for a total of three cycles.

Systemic treatment followed routine first-line NSCLC practice. Pembrolizumab monotherapy was administered for tumors with a PD-L1 tumor proportion score (TPS) ≥50%, and pembrolizumab combined with platinum-based chemotherapy was used for PD-L1 TPS <50%. Patients assigned to the control arm received the same systemic treatment algorithm without preceding cryotherapy. If the cryotherapy procedure could not be technically completed, the patient was managed on the control treatment pathway for subsequent analysis.

### 2.2. Radiological Assessment

Tumor response was assessed by contrast-enhanced computed tomography every three treatment cycles and categorized according to Response Evaluation Criteria In Solid Tumors (RECIST) version 1.1 as partial response, stable disease, or progressive disease. Cases that were not straightforward on routine radiologic review were discussed in a multidisciplinary tumor board.

### 2.3. Blood Collection, Processing, and Flow Cytometry

Peripheral blood was collected in EDTA tubes at three predefined time points: baseline, week 3, and week 6. In the cryotherapy arm, the baseline sample was obtained immediately before bronchoscopy; in the control arm, and at follow-up time points in both groups, blood was drawn before systemic treatment administration. Peripheral blood mononuclear cells were isolated from fresh blood by density-gradient centrifugation and analyzed without cryopreservation.

**Figure 1:**
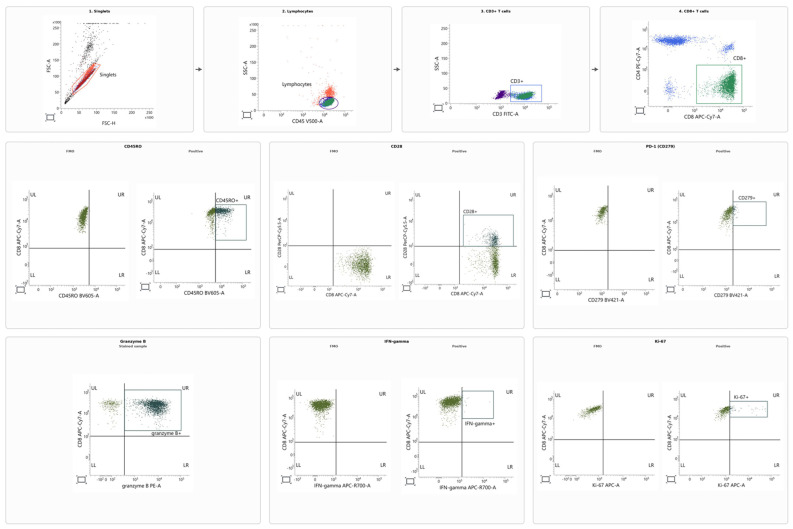
Flow-cytometry gating strategy for peripheral blood CD8+ T cell subsets. After selection of singlets and lymphocytes and subsequent identification of the CD8+ compartment, gates were applied for CD45RO, CD28, granzyme B, Interferon-γ, Ki-67, and PD-1 (CD279). Fluorescence-minus-one controls were used to support threshold definition for CD45RO, CD28, Interferon-γ, Ki-67, and PD-1.

Flow cytometry was performed for CD8, CD45RO, CD28, PD-1 (CD279), granzyme B (GzB), Interferon-γ (IFNγ), and Ki-67 markers. Fluorochromes used for cell identification were CD8 (APC-Cy7, clone SK1, BD Biosciences, Franklin Lakes, NJ, USA, Cat. No. 557834), CD45RO (BV605, clone UCHL1, BD Biosciences, Cat. No. 562791), CD28 (PerCP-Cy5.5, clone L293, BD Biosciences, Cat. No. 337181), PD-1/CD279 (BV421, clone MIH4, BD Biosciences, Cat. No. 564323), Granzyme B (PE, clone GB11, BD Biosciences, Cat. No. 561142), IFNγ (APC-R700, clone B27, BD Biosciences, Cat. No. 564981), and Ki-67 (APC, clone Ki-67, BioLegend, San Diego, CA, USA, Cat. No. 350514). As the PD-1 antibody, used in this panel, only detects accessible PD-1 epitopes under the staining conditions used, and all patients received pembrolizumab after baseline sampling, post-treatment PD-1+, CD28+PD-1+, GzB+PD-1+, and PD-1+Ki-67+ results were interpreted as changes in detectable PD-1 staining and epitope saturation with pembrolizumab, rather than changes in expression. Surface markers were stained before fixation, and intracellular targets were assessed after fixation/permeabilization according to the manufacturer’s protocol. Representative gating plots are shown in Figure 1.

For statistical analysis, CD3+ T cells were expressed as a percentage of all lymphocytes, whereas CD4+ and CD8+ cells were expressed as percentages of CD3+ T cells. CD45RO+, CD28+, GzB+, IFNγ+, PD-1+, Ki-67+, and PD-1+Ki-67+ populations were expressed as percentages of CD8+ T cells. CD28+ Ki-67+ and CD28+ PD-1+ were calculated within CD28+ cells, and GzB+ Ki-67+ and GzB+ PD-1+ within GzB+ cells. Data was analyzed using BD FACSuite software version 1.5.

### 2.4. Outcomes

The immunologic outcomes of this analysis were longitudinal changes in circulating CD8+ T cell subsets from baseline to week 3 and week 6, and differences in these changes between the cryotherapy and control groups. Additionally, subgroup analyses according to radiologic response and the association of CD8+ changes during treatment with progression-free survival (PFS) and overall survival (OS) were performed.

A formal a priori power calculation was not performed. The study was designed as an exploratory analysis because reliable preliminary estimates of the expected magnitude of bronchoscopic cryotherapy-induced CD8+ T cell changes in metastatic NSCLC were not available. The sample size was based on feasibility and the expected number of eligible patients during the study period. The immune analyses were mainly interpreted as exploratory and are presented with effect estimates and 95% confidence intervals. Survival analyses were considered hypothesis-generating because of the modest number of patients, attrition, and multiple biomarkers tested.

PFS and OS were evaluated using an as-treated (modified intention-to-treat) analysis to better reflect the clinical impact of the immunological changes induced by cryotherapy. For translational analyses, only patients with available blood samples at the relevant time point were included. No imputation was applied for missing flow-cytometry data; analyses were performed on an available-case basis.

### 2.5. Statistical Analysis

Continuous variables are presented as medians with interquartile ranges, and categorical variables as counts and percentages. Inferential analyses of longitudinal immune changes were performed using mixed-effects models, and model-derived estimates are reported as adjusted percentage-point differences with 95% confidence intervals (CIs). Time was modeled as a categorical variable with three levels (baseline, week 3, and week 6). For the primary treatment-group analysis, each immune subset was analyzed in a separate model including fixed effects for treatment group (cryotherapy vs. controls), time, treatment group-by-time interaction, and systemic regimen (pembrolizumab monotherapy vs. pembrolizumab with platinum-based chemotherapy), with a patient-specific random intercept to account for within-patient correlation across repeated measurements. Within-group changes from baseline were estimated using model-derived marginal means and pairwise contrasts. For subsets showing right-skewed residuals, log-transformed sensitivity analyses were performed. These included CD28+Ki-67+, CD28+PD-1+, GzB+Ki-67+, IFNγ+, Ki-67+, and PD-1+Ki-67+ subsets. For radiologic response analyses, patients with partial response (PR) were classified as responders and those with stable (SD) or progressive disease (PD) as non-responders. Radiologic response analyses were performed using mixed-effects models. Response-associated immune trajectories were analyzed using models including response group (responders vs. non-responders), time, the response group-by-time interaction, treatment group, and systemic regimen, with patient-specific random intercepts. Additional exploratory subgroup analyses were performed separately within the cryotherapy and control groups.

PFS and OS were estimated using the Kaplan–Meier method and compared using the log-rank test. For analyses of post-treatment immune variables, landmark Cox proportional hazards models were used to reduce immortal-time bias. Week 3 analyses used a 21-day landmark, and week 6 analyses used a 42-day landmark. Patients who had experienced the relevant event or were censored before the landmark, or who lacked the required paired immune measurements, were excluded from the corresponding landmark model. Survival time was recalculated from the landmark date. CD8 T cell dynamics were entered as continuous log2-transformed week 3-to-baseline or week 6-to-baseline ratios, so that hazard ratios (HRs) represent the association per doubling of the landmark/baseline ratio. Each marker was analyzed in a separate Cox model. The primary multivariable model included the CD8 T cell subpopulation, treatment group, and (PD-L1) tumor proportion score category (<1%, 1–49%, ≥50%), with PD-L1 <1% used as the reference category. Additional exploratory sensitivity models adjusted for age and gender, and separately for liver and brain metastases. Broader multivariable models were avoided due to the relatively low number of events. Survival analyses were considered exploratory because of sample size, attrition at post-treatment landmarks, and multiple biomarker testing. The proportional-hazards assumption was assessed using the Schoenfeld residuals test. No formal multiplicity correction was applied because the study was exploratory and hypothesis-generating. All tests were two-sided, and a *p*-value < 0.05 was considered statistically significant. Statistical analyses were performed using SPSS version 30.0.0.0 and plotted using GraphPad Prism version 10.6.1.

## 3. Results

### 3.1. Patient Characteristics

**Figure 2:**
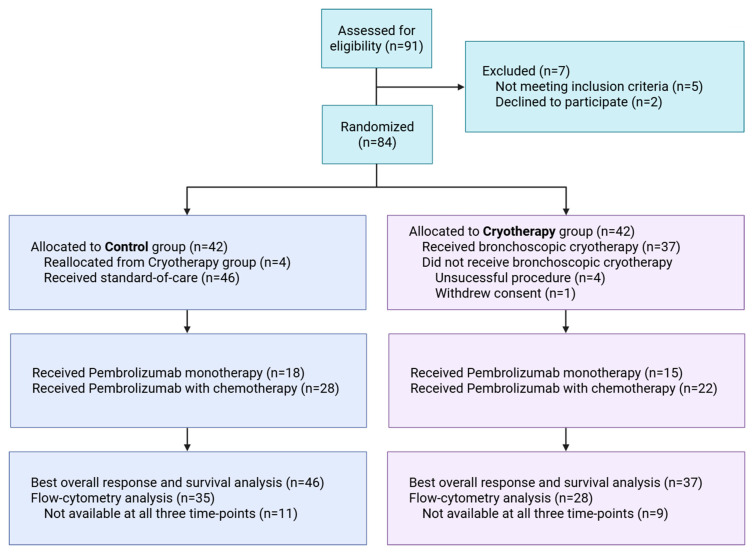
CONSORT diagram.

As described in our earlier paper, flow cytometry was performed for 76 patients, with 34 in the cryotherapy group and 42 in the control group, with baseline demographic and disease characteristics included (Figure 2) [14]. Four patients, initially assigned to the cryotherapy group, were considered post-randomization intercurrent events and reassigned to the control group due to an unsuccessful procedure.

At baseline, peripheral blood samples were available for flow cytometry analysis in all patients. Due to attrition, 30 cryotherapy patients could provide samples at week 3 and at weeks 28–6. In the control group, 35 patients provided samples at weeks 3 and 6. This led to landmark survival populations of 65 and 63 patients for week 3 and 6 models, respectively.

### 3.2. Changes in Peripheral Blood T Cells and CD8+ Subsets During Treatment

**Figure 3:**
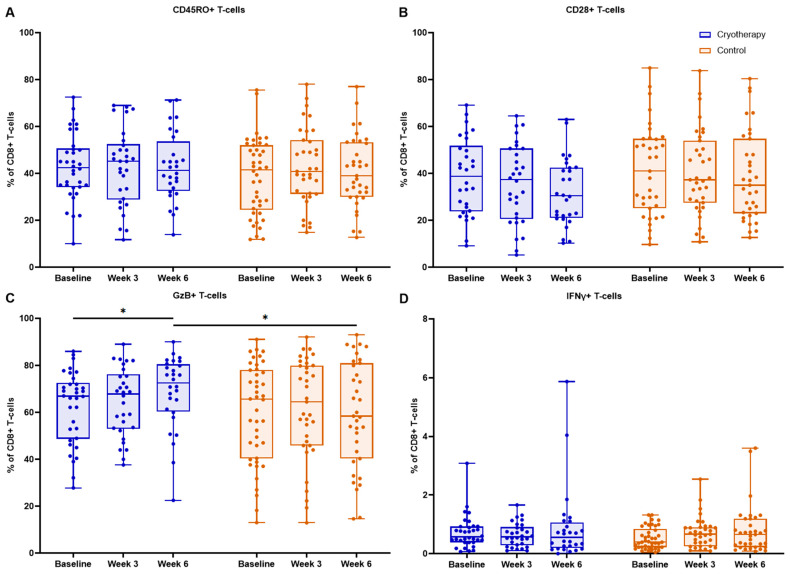
Dynamics of peripheral blood (**A**) CD45RO+, (**B**) CD28+, (**C**) GzB+, and (**D**) IFNγ T cells in cryotherapy and control groups at baseline, week 3, and week 6. Boxes indicate median and interquartile range; points represent individual patients; * *p* < 0.05. Only significant changes that were retained in the log-transformed sensitivity analysis are marked.

As described in our earlier paper, a significant longitudinal shift was observed for both CD4+ and CD8+ T cell populations in the cryotherapy group, with no significant changes in controls [14]. Observed values and within-group changes for CD8+ T cell subsets are provided in the Supplemental Table S1. In the cryotherapy group, the most consistent CD8+ T cell change was an increase in circulating GzB+ CD8+ T cells from baseline to week 6 (Figure 3C). The observed median proportion increased from 66.91% (49.89–72.00) at baseline to 72.52% (61.01–80.09) at week 6. The model-derived within-group change was +5.39 percentage points (95% CI 1.68 to 9.10; *p* = 0.004). Interestingly, CD28+ CD8+ T cells decreased in the cryotherapy group by week 6, from 38.73% (24.40–50.46) at baseline to 30.44% (21.67–42.43) at week 6, with an adjusted change of −9.70 percentage points (95% CI −15.14 to −4.26; *p* < 0.001) (Figure 3B). No significant changes were observed in the CD45RO+ CD8+ and IFNγ+ CD8+ populations in the cryotherapy group, and no aforementioned population changes were observed in the controls (Figure 3A–D).

**Figure 4:**
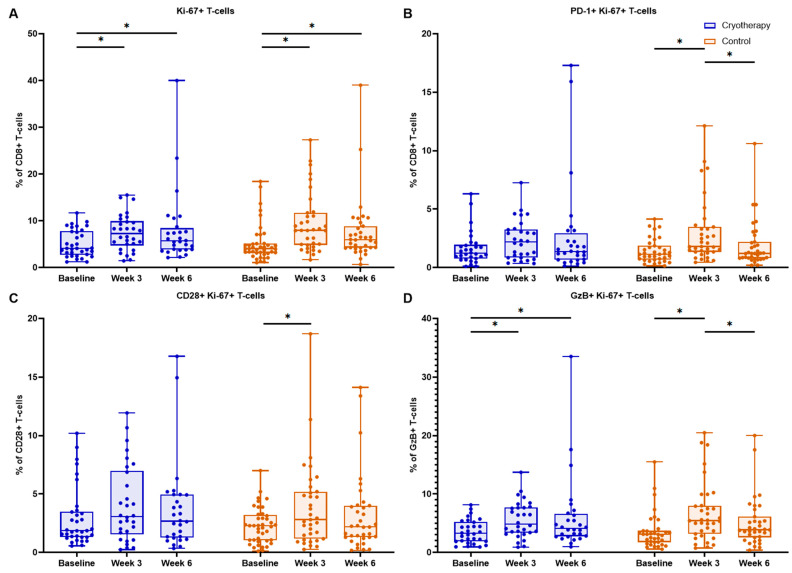
Dynamics of peripheral blood (**A**) Ki-67+, (**B**) PD-1+ Ki-67+, (**C**) CD28+ Ki-67+, and (**D**) GzB+ Ki-67+ T cells in cryotherapy and control groups at baseline, week 3, and week 6. Boxes indicate median and interquartile range; points represent individual patients; * *p* < 0.05. Only significant changes that were retained in the log-transformed sensitivity analysis are marked.

CD8+ T cell proliferation was observed in both treatment groups (Figure 4A). In the cryotherapy group, Ki-67+ CD8+ T cells increased from 4.08% (2.89–7.65) at baseline to 7.24% (5.01–9.72) at week 3 and remained elevated at 5.65% (3.84–7.92) at week 6. In the mixed-effects model, this led to increases from baseline at week 3 (+2.35 percentage points, 95% CI 0.02 to 4.67; *p* = 0.048) and from baseline to week 6 (+2.90 percentage points, 95% CI 0.52 to 5.27; *p* = 0.017). In the control group, Ki-67+ CD8+ T cells increased from 4.03% (3.13–5.08) at baseline to 7.94% (4.87–11.36) at week 3 and 5.90% (4.24–8.62) at week 6. Changes from baseline to weeks 3 and 6 were +4.39 percentage points (95% CI 2.26 to 6.52; *p* < 0.001) and +2.49 percentage points (95% CI 0.36 to 4.62; *p* = 0.022), respectively. The Ki-67+ changes remained significant after log-transformed sensitivity analyses.

Similar increases were observed for the GzB+ Ki-67+ subset, which increased from 3.24% (1.92–4.99) at baseline to 4.83% (3.42–7.65) at week 3 and 4.09% (2.78 6.44) at week 6 in the cryotherapy group (Figure 4D). The adjusted changes from baseline to weeks 3 and 6 were +2.14 percentage points (95% CI 0.18 to 4.10; *p* = 0.032) and +2.53 percentage points (95% CI 0.55 to 4.52; *p* = 0.012), respectively. In the control group, GzB+Ki-67+ cells also increased from 3.06% (1.75–3.67) at baseline to 5.41% (3.42–7.96) at week 3, by +3.14 percentage points (95% CI 1.34 to 4.94; *p* < 0.001). However, this was followed by a decrease of −2.22 percentage points from week 3 to week 6 (95% CI −4.07 to −0.37; *p* = 0.018), to 3.85% (2.61–5.74). These GzB+ Ki-67+ findings were also supported by log-transformed sensitivity analyses.

PD-1+ Ki-67+ CD8+ cells in the cryotherapy group increased from 1.21% (0.81–1.86) at baseline to 1.36% (0.69–2.48) at week 6, although this result was not retained after log transformation and should be interpreted cautiously (Figure 4B). Similarly, PD-1+ Ki-67+ cells in the control group showed a transient pattern, increasing from 1.13% (0.70–1.79) at baseline to 1.79% (1.33–3.38) at week 3 (+1.56 percentage points, 95% CI 0.60 to 2.51; *p* = 0.001) and decreasing to 1.20% (0.81–2.16) at week 6 (−1.06 percentage points, 95% CI −2.01 to −0.11; *p* = 0.028).

**Figure 5:**
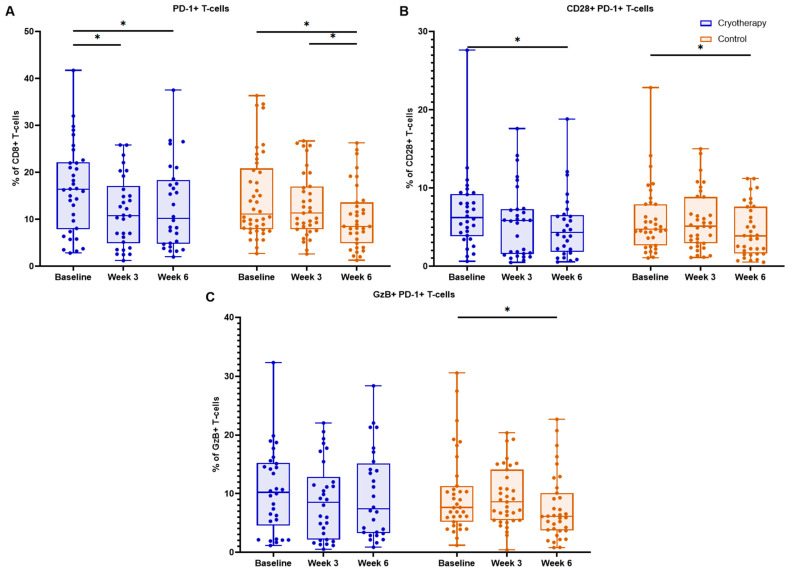
Dynamics of peripheral blood (**A**) PD-1+, (**B**) CD28+ PD-1+, and (**C**) GzB+ PD-1+ T cells in cryotherapy and control groups at baseline, week 3, and week 6. Boxes indicate median and interquartile range; points represent individual patients; * *p* < 0.05. PD-1-associated populations represent detectable antibody-accessible epitope staining and may have been influenced by pembrolizumab administration. Only significant changes that were retained in the log-transformed sensitivity analysis are marked.

Exhaustion-associated markers decreased over time in both groups (Figure 5A–C). In the cryotherapy group, PD-1+ CD8+ T cells decreased from 16.37% (8.46–22.00) at baseline to 10.79% (5.40–16.03) at week 3 and 10.16% (4.84–17.84) at week 6. This resulted in a decrease of −4.80 percentage points from baseline to week 3 (95% CI −7.64 to −1.96; *p* < 0.001) and −4.01 percentage points by week 6 (95% CI −6.92 to −1.10; *p* = 0.007). In the control group, PD-1+ CD8+ cells decreased from 11.08% (7.97–20.33) at baseline to 8.50% (5.32–13.18) at week 6, with an overall change of −4.11 percentage points (95% CI −6.73 to −1.50; *p* = 0.002). CD28+ PD-1+ cells decreased by week 6 in both groups, and GzB+ PD-1+ cells decreased by week 6 in controls (Figure 5B,C). As all patients received pembrolizumab-based therapy, decreases in PD-1 staining should be interpreted as reduced detectable antibody-accessible epitopes due to therapeutic pembrolizumab binding, rather than a direct reduction in total PD-1 protein expression.

### 3.3. Cryotherapy Associated Changes in Peripheral CD8+ Subsets

As within-group changes do not directly establish a treatment-specific effect, we next evaluated CD8+ T cell changes induced by cryotherapy. The clearest longitudinal signal, specific to cryotherapy, was observed for GzB+ CD8+ T cells. The increase in this subset was greater in the cryotherapy group than in controls, with an adjusted between-group difference in change of +5.33 percentage points (95% CI 0.34 to 10.32; *p* = 0.036). A significant between-group difference was also observed for the week 3 to week 6 interval (+5.03 percentage points, 95% CI 0.01 to 10.05; *p* = 0.049) (Figure 3C, Supplemental Table S2).

In contrast, proliferation-associated changes were not specific to the cryotherapy group alone. Ki-67+ CD8+ and GzB+ Ki-67+ subsets increased within both treatment arms, and treatment group-by-time interaction did not support a significantly greater increase in cryotherapy patients. These findings suggest that early peripheral CD8+ proliferation was more likely related to pembrolizumab-based systemic therapy than to cryotherapy itself.

For PD-1+ Ki-67+ CD8+ cells, the original model showed a significant benefit of cryotherapy in change from week 3 to week 6 (+1.56 percentage points, 95% CI 0.14 to 2.98; *p* = 0.031). However, because this subset showed residual skewness and the interaction was not significant in the log-transformed sensitivity analysis, this finding should be considered more cautiously than the GzB+ CD8+ change.

### 3.4. CD8+ T Cell Changes According to Radiologic Response

Among the 76 evaluable patients, 21 achieved a partial response and were classified as responders, whereas 55 patients with stable or progressive disease were classified as non-responders, with the cryotherapy group having a significantly higher overall response rate than controls, as described in an earlier paper [14]. Across the full study group, responders were associated with more sustained proliferative CD8+ dynamics.

Ki-67+ CD8+ T cells showed a greater increase from baseline to week 6 in responders than non-responders at +4.25 percentage points (95% CI 0.86 to 7.63; *p* = 0.014) (Table 1). However, this finding weakened after log transformation. More robust findings were observed for CD28+ Ki-67+ cells. Responders showed a greater increase from baseline to week 6 than non-responders, with an adjusted between-group difference of +2.68 percentage points (95% CI 1.07 to 4.30; *p* = 0.001), and this finding remained significant in log-transformed sensitivity analysis. No significant differences between responders and non-responders were observed for other T cell populations; however, several borderline changes were observed, mainly in GzB+ Ki-67+, IFNγ+, and PD-1+Ki-67+.

We further explored differences in CD8+ T cell populations between responders and non-responders separately within the cryotherapy and control groups. While these subgroup analyses should be interpreted more cautiously because of limited sample sizes, they appeared to reflect the overall study cohort. Within the cryotherapy group, responders showed stronger evidence of sustained immune activation than non-responders, with CD28+ Ki-67+ cells increasing by +3.78 percentage points from baseline to week 6 (95% CI 1.33 to 6.24; *p* = 0.003), compared to non-responders (Table 2). A significant difference was also observed from week 3 to week 6 (+2.90 percentage points, 95% CI 0.41 to 5.38; *p* = 0.022), although this interval-specific finding was not retained after log transformation. Cryotherapy responders also showed a greater increase from baseline to week 6 in IFNγ+ CD8+ cells than non-responders (+1.16 percentage points, 95% CI 0.39 to 1.92; *p* = 0.003), with a log-transformed sensitivity model remaining significant. In addition, CD28+ CD8+ cells showed a greater increase from baseline to week 3 in cryotherapy responders than non-responders (+10.96 percentage points, 95% CI 0.28 to 21.63; *p* = 0.044), although this finding should be interpreted cautiously, considering the borderline statistical significance and the apparent discrepancy to the whole cryotherapy group dynamic, mentioned earlier. In the control group, responder numbers were small (n = 7). No differences in CD8+ T cell changes reached statistical significance in the control group between responders and non-responders (Table 3).

### 3.5. Correlation of Peripheral Blood CD8+ T Cell Profiles with Clinical Outcomes

We next explored whether observed CD8+ T cell changes were associated with clinical outcomes using Cox proportional-hazards models. Landmark population details are provided in the Supplemental Table S3.

In the week 3 landmark analysis, the most consistent exploratory T cell signal, related to survival, was the GzB+ Ki-67+T cell change. A higher week 3 to baseline GzB+ Ki-67+ ratio was associated with longer PFS in univariable analysis (*p* = 0.012) and remained significant after adjustment for treatment group and PD-L1 expression (HR 0.72 per doubling, 95% CI 0.54–0.96; *p* = 0.024) (Table 4). A similar, although borderline, association was observed for OS (adjusted HR 0.72, 95% CI 0.52–0.99; *p* = 0.046). After additional sensitivity adjustment for age and gender, in addition to treatment group and PD-L1 expression, the week 3 GzB+ Ki-67+ ratio remained associated with both PFS (HR 0.72, 95% CI 0.54–0.96; *p* = 0.026) and OS (HR 0.71, 95% CI 0.51–0.99; *p* = 0.044). Additional sensitivity adjustment for liver and brain metastases attenuated the week 3 GzB+ Ki-67+ association (PFS HR 0.79, 95% CI 0.58–1.07; *p* = 0.132; OS HR 0.73, 95% CI 0.51–1.04; *p* = 0.083); however, a broader multivariable adjustment was limited by the event count, making the model more unstable. These findings identify week-3 GzB+ Ki-67+ expansion as a possible candidate survival biomarker.

The week 3 Ki-67+ CD8+ ratio showed a directionally favorable association with PFS and OS, but did not reach statistical significance after adjustment. Meanwhile, a higher week 3 PD-1+ Ki-67+ ratio was associated with longer PFS (adjusted HR 0.78, 95% CI 0.61–0.99; *p* = 0.039).

In the week 6 landmark analysis, no CD8+ T cell ratio showed a robust association with PFS or OS after adjustment for treatment group and PD-L1 category. CD45RO+ showed borderline associations with both PFS and OS, but these did not meet significance thresholds (Supplemental Table S4).

## 4. Discussion

In this exploratory prospective randomized study of metastatic NSCLC patients receiving first-line pembrolizumab-based therapy, the addition of bronchoscopic cryotherapy was associated with a distinct remodeling of the peripheral CD8+ T cells towards enhanced cytotoxic activity, as an increase in the GzB+ CD8+ population. Meanwhile, elevated proliferation of total CD8+ T cells was observed in both groups and appeared to be more closely related to the effect of immunotherapy itself. While comparison of flow cytometry results with radiological response revealed few correlations present in both the overall study population and cryotherapy or control subgroups, the proliferation of CD8+ T cells and the CD28+ subset was more prevalent and sustained in responders. These two changes, in particular, translated into better radiologic outcomes, with responders presenting a consistent increase in the aforementioned populations. Meanwhile, only the proliferation of cytotoxic CD8+ T cells consistently led to increased survival. In exploratory landmark survival analyses, GzB+ Ki-67+ expansion by week 3 showed a hypothesis-generating association with longer PFS and OS, although the modest event count, multiple biomarker testing, borderline OS significance, and attenuation after additional adjustment for liver and brain metastases warrant a cautious interpretation. Several pathways have been observed to link cryotherapy and improved CD8+ T cell infiltration with higher effector and cytolytic activity in preclinical murine lung cancer models [10,15,16]. At the same time, the available literature indicates that immunity induced via cryotherapy alone is not durable, and the early phase of immune stimulation may be countered by regulatory suppression. In the bilateral breast tumor model reported by Yu et al., cryoablation initially increased tumor-infiltrating CD45+, CD4+, and CD8+ cells and reduced Tregs and myeloid-derived suppressor cells, but this favorable state was later accompanied by upregulation of PD-1/PD-L1 signaling and a shift toward a more suppressive microenvironment [17]. However, the addition of immune checkpoint blockade improves tumor control beyond either monotherapy by rescuing functionally exhausted effector cells [11,17,18].

Studies translating these models into clinical settings are still limited and heterogeneous in their methods. Gu et al. examined six patients with early-stage NSCLC and observed a significant increase in circulating CD8+ T cells after cryoablation [11]. Furthermore, paired biopsies from three patients with unresectable NSCLC treated with cryoablation plus anti-PD-1 therapy showed increased intratumoral CD8+ infiltration. A similar signal was observed in the CRYOVATE study [9]. 8 patients with previously untreated advanced NSCLC and PD-L1 expression of 50% or greater were enrolled to receive cryotherapy, followed by pembrolizumab monotherapy. Post-treatment biopsies from patients with clinical benefit showed increased CD8+ T cell infiltration compared with those without clinical benefit, with a significant difference in post-treatment CD8+ levels between the two groups. Furthermore, a phase I bronchoscopic cryotherapy study of 21 advanced NSCLC patients by Tsay et al. observed a shift in the peripheral blood away from naïve CD8+ cells toward proliferating Ki-67+ CD8+ T cells as early as day 14 after the procedure [8]. Together, these studies show that cryotherapy may induce significant changes in the cancer microenvironment, which are mirrored in the peripheral blood. Data from other cancers suggest that the effect of cryotherapy on CD8+ T cells is not unique to lung cancer. In renal cancer, Kato et al. analyzed paired pre- and post-cryoablation tumor tissues from 22 patients and found increased expression of CD8, CD4, granzyme A, and CD11c after cryoablation, together with a higher CD8/FOXP3 ratio and evidence of oligoclonal expansion of circulating anti-tumor T cells [19].

Meanwhile, studies of immunotherapy alone in cancer patients, including NSCLC, have highlighted several CD8+ T cell subsets, which appear to be affected. Ki-67 is a marker of cell proliferation [20]. Immunotherapy has been shown to increase CD8+ T cell proliferation, as well as an increase in Ki-67+ T cells, predicting a favorable response in NSCLC patients [21,22,23]. While we observed similar results, with Ki-67+ CD8+ T cell populations increasing in both treatment groups, this increase was not a significant positive predictor for both PFS and OS in a multivariable analysis. However, responders appeared to show a greater increase in Ki-67+ CD8+ T cells, compared to non-responders. On the opposite end of the T cell activation is PD-1, which represents exhaustion from persistent antigen stimulation [24,25]. Anti-PD-1 Immune checkpoint inhibitors (ICIs), such as pembrolizumab, bind to this molecule to reinvigorate the anti-tumor immune response [26]. Several studies observed increased PD-1+ CD8+ T cell proliferation, following immunotherapy [24,27]. Meanwhile, we observed decreases in this population in both groups, with the change happening earlier in the cryotherapy group. However, we used anti-PD-1 antibodies in our analysis, which would compete with pembrolizumab for the same T cell receptor [24]. Therefore, the PD-1+ T cell dynamics in both therapy groups should be viewed through the lens of these cells being saturated with pembrolizumab antibodies.

CD45RO+ CD8+ cells broadly reflect the memory T cells; further division is possible [28]. For example, in advanced melanoma patients receiving ipilimumab or pembrolizumab immunotherapy, higher peripheral blood levels of CD45RO+ CD8+ were indicative of response to immunotherapy [29]. However, this effect was localized to patients receiving ipilimumab, an anti-CTLA-4 ICI, and no clinical benefit was observed in patients receiving pembrolizumab, which correlates with our findings. However, a subset of memory T cells, namely central memory T cells, appears to correlate with improved survival in NSCLC and melanoma patients receiving nivolumab, providing an impetus for further research on these cells [30]. In our study, CD45RO+ proportions remained relatively stable, suggesting that either the dominant early signal after cryotherapy or immunotherapy was not in this subset, or a deeper examination is required.

Another analyzed subset, CD28+ cells, preserves co-stimulatory capacity and is generally considered less terminally differentiated and more responsive to reactivation [31,32]. CD28 expression was shown to be essential for CD8+ T cell proliferation, which appears predominantly in the CD28+ subset after PD-1 blockade in lung cancer patients [24]. In peripheral blood of melanoma, NSCLC, and pancreatic ductal adenocarcinoma patients, PD1+ CD28+ T cells appear functionally inferior to PD1+ CD28− T cells; however, this tendency was reversed within tumor tissues, where preserved CD28 expression was associated with maintained effector function [33]. In a clinical context, higher baseline peripheral blood CD28+ CD8+ T cells were associated with improved survival in patients receiving PD-1/PD-L1 inhibitors across several cancers [34]. While in our study, total peripheral CD28+ CD8+ T cells decreased in the cryotherapy group, but this change did not differ compared to controls. An interesting dynamic was revealed by a further analysis. Both treatment groups presented a decrease in CD28+ PD-1+ cells. As this subset was described as functionally inferior in other studies, we expected similar results [31,33]. However, PD-1 expression on CD28+ T cells in our study appeared to have no significant impact on radiological response across the whole study population, showing significance; however, in the cryotherapy group, this might be impacted by a small sample size. In contrast, elevated Ki-67 expression on CD28+ appeared to be more closely related to treatment outcomes, as was observed in responders only.

The cytotoxic and effector markers in our panel appear to best refine our interpretation of cryotherapy effects. GzB is a core marker of CD8+ T cell-mediated tumor cell killing [35]. Pembrolizumab immunotherapy in metastatic melanoma increases GzB+ CD8+ T cell infiltration into tumor tissue, a sign of enhanced effector response, although these changes appear to be more prevalent in patients with radiological response [36]. Furthermore, NSCLC patients with higher serum levels of granzyme B exhibit higher PFS and OS [37]. In our study, GzB+ CD8+ T cells increased in the cryotherapy group, suggesting enhanced cytotoxic readiness after local tumor destruction. The GzB+ Ki-67+ subset appeared to be particularly informative due to the significant correlation with PFS and OS, although its correlation with radiological response was less robust. We also observed measurable decreases in GzB+ PD-1+ cells, mainly in the control group. This did not translate into a significant clinical response, in contrast to the findings by Chung et al., who observed higher levels of GzB+ PD-1+ CD8+ T cells in NSCLC patients with durable clinical benefit after 3 weeks of pembrolizumab immunotherapy [38]. However, this effect was observed in patients with PD-L1 TPS of 1% or higher and may be impacted by the heterogeneity in our study population. IFNγ reflects effector cytokine function and a broader inflammatory response of CD8+ T cells [35,39]. However, the duality of IFNγ is reflected in both the anti-tumor immune response, as well as the immune evasion by cancer cells, especially by increased PD-L1 expression [40,41]. In metastatic melanoma patients receiving pembrolizumab or ipilimumab/nivolumab, IFNγ receptor β chain expression in CD8+ T cells was negatively correlated with clinical response [42]. Meanwhile, we did not observe significant changes in IFNγ+ CD8+ T cells. Combined with earlier studies, this result would suggest that T cell sensitivity to IFNγ, rather than the expression of IFNγ itself in the peripheral blood, is a better predictor for response.

To our knowledge, no other studies have evaluated the peripheral CD8+ T cell dynamics during treatment as predictors of clinical outcomes in cancer patients receiving cryotherapy combined with immunotherapy. However, our findings, combined with preclinical data, present this treatment as a potentially improved anti-tumor response. However, as our study was mainly exploratory, further research is needed to ascertain this hypothesis.

Our study has several limitations. First, the relatively small sample size, together with attrition during serial blood collection, may have reduced the power to detect significant differences across CD8+ T -cell subsets, particularly in subgroup analyses. This was most evident among patients with progressive disease and among responders in the control group, whose low numbers limit firm interpretation. Second, although our panel captured selected markers of memory, co-stimulation, cytotoxicity, proliferation, and exhaustion, it did not provide deeper phenotypic or functional resolution of CD8+ T cell states, such as detailed naïve, effector, stem-like, or terminally exhausted subsets. Third, the analysis relied on peripheral blood immunophenotyping without paired tumor sampling, limiting conclusions about whether circulating changes directly reflected intratumoral immune remodeling after cryotherapy. Fourth, the interpretation of PD-1-associated populations was limited by potential pembrolizumab-mediated epitope masking. Fifth, no formal multiplicity correction was applied across the immune-marker panel. Therefore, statistically significant biomarker associations, particularly survival associations, should be regarded as exploratory and hypothesis-generating. In addition, heterogeneity in systemic treatment, including pembrolizumab monotherapy versus chemoimmunotherapy, as well as variation in PD-L1 expression, may have influenced immune dynamics despite randomization and require further investigation.

## 5. Conclusions

In metastatic NSCLC patients receiving first-line pembrolizumab-based therapy, the addition of bronchoscopic cryotherapy was associated with a distinct peripheral CD8+ T cell remodeling characterized mainly by increased cytotoxic readiness, reflected by expansion of the GzB+ CD8+ compartment. Proliferative CD8+ T cell changes occurred in both treatment arms, suggesting that they were largely shared effects of pembrolizumab-based systemic therapy. Clinical outcomes were associated with enhanced proliferative activity, with CD28+ Ki-67+ CD8+ T cells predicting better radiological response and GzB+ Ki-67+ CD8+ T cells possibly leading to longer PFS and OS. These findings support the hypothesis that bronchoscopic cryotherapy is a potential immune-modulating adjunct and identify longitudinal changes in the proliferation of cytotoxic CD8+ T cells as candidate biomarkers requiring validation in larger studies.

## Figures and Tables

**Table 1:** Peripheral blood CD8+ T cell changes according to radiological response.

Population	Responders			Non-Responders			Difference in Change (95% CI)		
	Baseline (n = 21)	Week 3 (n = 21)	Week 6 (n = 21)	Baseline (n = 55)	Week 3 (n = 44)	Week 6 (n = 42)	Baseline to Week 3	Baseline to Week 6	Week 3 to Week 6
CD45RO+ (% of CD8+)	41.97 (31.36–49.78)	40.48 (31.50–53.56)	40.00 (33.75–45.64)	42.15 (29.04–52.09)	45.70 (30.93–53.50)	40.81 (32.00–53.20)	−1.39 (−8.88 to +6.10)	+2.99 (−4.54 to +10.51)	+4.37 (−3.21 to +11.95)
CD28+ (% of CD8+)	33.11 (23.33–47.10)	38.00 (29.85–51.97)	31.50 (22.43–42.59)	44.62 (25.91–54.88)	36.38 (25.42–50.49)	34.22 (21.98–47.90)	+6.27 (−2.72 to +15.26)	+0.86 (−7.43 to +9.15)	−5.41 (−14.11 to +3.29)
CD28+ Ki-67+ (% of CD28+)	1.75 (1.22–2.64)	3.00 (1.72–5.20)	4.04 (1.42–4.95)	2.29 (1.13–3.67)	2.87 (1.15–5.76)	2.21 (1.25–3.73)	+1.21 (−0.39 to +2.82)	**+2.68 (+1.07 to +4.30)**	+1.47 (−0.15 to +3.09)
CD28+ PD-1+ (% of CD28+)	6.01 (3.91–8.03)	5.92 (1.60–10.16)	3.25 (1.07–8.22)	5.13 (3.56–8.28)	5.06 (2.35–6.55)	4.00 (2.23–6.33)	+0.63 (−1.53 to +2.79)	−0.30 (−2.41 to +1.82)	−0.93 (−3.10 to +1.24)
GzB+ (% of CD8+)	69.10 (52.83–76.00)	70.49 (53.58–78.94)	74.04 (57.86–79.03)	65.42 (43.19–75.00)	61.65 (49.33–77.71)	66.00 (46.80–80.75)	−1.18 (−6.54 to +4.19)	+0.33 (−5.07 to +5.72)	+1.50 (−3.90 to +6.91)
GzB+ Ki-67+ (% of GzB+)	2.42 (1.75–3.45)	4.97 (3.61–7.64)	3.87 (2.81–6.70)	3.18 (1.95–5.42)	5.46 (3.16–7.93)	3.96 (2.68–5.95)	+1.17 (−1.64 to +3.97)	+2.69 (−0.16 to +5.53)	+1.52 (−1.33 to +4.37)
GzB+ PD-1+ (% of GzB+)	10.68 (7.40–15.61)	9.52 (2.90–14.84)	6.54 (2.85–13.48)	7.26 (4.39–13.37)	7.13 (5.04–11.59)	6.14 (3.85–11.10)	−1.05 (−4.26 to +2.16)	−2.17 (−5.36 to +1.01)	−1.12 (−4.36 to +2.11)
IFNγ+ (% of CD8+)	0.52 (0.23–0.79)	0.74 (0.53–0.94)	0.72 (0.26–1.19)	0.46 (0.28–0.94)	0.55 (0.24–0.88)	0.41 (0.23–0.89)	+0.26 (−0.20 to +0.72)	+0.46 (−0.01 to +0.92)	+0.20 (−0.27 to +0.66)
Ki-67+ (% of CD8+)	3.46 (3.18–4.37)	7.89 (4.80–9.76)	5.75 (4.26–7.54)	4.30 (2.90–7.42)	7.93 (4.99–10.83)	6.01 (4.04–8.62)	+1.44 (−1.93 to +4.81)	**+4.25 (+0.86 to +7.63)** †	+2.81 (−0.62 to +6.23)
PD-1+ (% of CD8+)	14.88 (9.73–19.33)	12.46 (3.90–19.22)	8.10 (3.80–17.31)	13.66 (7.71–22.30)	10.19 (7.00–16.02)	9.66 (6.07–15.02)	−0.81 (−5.02 to +3.40)	−1.52 (−5.75 to +2.71)	−0.71 (−4.97 to +3.55)
PD-1+ Ki-67+ (% of CD8+)	1.14 (0.84–1.48)	2.09 (0.83–3.31)	1.19 (0.66–1.83)	1.17 (0.72–2.22)	1.94 (1.15–3.30)	1.31 (0.86–2.98)	+0.53 (−0.97 to +2.03)	+1.44 (−0.06 to +2.95)	+0.91 (−0.59 to +2.42)

Values are medians (IQR). n—number of patients. Differences are adjusted for responder minus non-responder differences in change from the mixed-effects model. Positive estimates indicate a greater increase in responders. Bold values indicate *p* < 0.05. † indicates a statistically significant original contrast that was not retained in the log-transformed sensitivity analysis. CI—confidence interval.

**Table 2:** Peripheral blood CD8+ T cell changes according to radiological response in the cryotherapy group.

Population	Responders			Non-Responders			Difference in Change (95% CI)		
	Baseline (n = 14)	Week 3 (n = 14)	Week 6 (n = 14)	Baseline (n = 21)	Week 3 (n = 16)	Week 6 (n = 14)	Baseline to Week 3	Baseline to Week 6	Week 3 to Week 6
CD45RO+ (% of CD8+)	43.42 (32.27–49.89)	40.91 (28.36–52.98)	41.50 (36.33–45.48)	42.11 (34.80–52.23)	46.23 (32.04–50.50)	40.81 (26.83–53.66)	+0.22 (−8.71 to +9.14)	+7.25 (−1.89 to +16.38)	+7.03 (−2.18 to +16.24)
CD28+ (% of CD8+)	34.50 (23.89–42.84)	37.39 (24.54–50.98)	26.21 (20.95–42.54)	44.62 (26.25–57.59)	35.88 (20.63–46.30)	31.02 (22.23–40.24)	**+10.96 (+0.28 to +21.63)**	+4.48 (−5.88 to +14.84)	−6.48 (−17.06 to +4.11)
CD28+ Ki-67+ (% of CD28+)	1.66 (1.24–2.48)	2.85 (1.71–4.22)	3.06 (1.18–4.95)	2.36 (1.39–6.35)	4.07 (1.74–7.38)	2.67 (1.60–3.70)	+0.89 (−1.51 to +3.29)	**+3.78 (+1.33 to +6.24)**	**+2.90 (+0.41 to +5.38)**
CD28+ PD-1+ (% of CD28+)	6.22 (4.46–7.92)	5.97 (1.74–9.46)	3.55 (1.36–7.06)	6.33 (3.46–9.40)	4.69 (1.64–6.63)	4.88 (2.57–6.28)	+1.92 (−0.98 to +4.81)	+0.60 (−2.32 to +3.52)	−1.32 (−4.28 to +1.64)
GzB+ (% of CD8+)	69.55 (59.10–73.87)	71.47 (54.19–77.63)	75.97 (66.01–80.28)	64.09 (47.25–70.78)	61.60 (53.01–69.51)	70.05 (60.34–79.37)	−1.31 (−9.51 to +6.90)	−0.80 (−9.19 to +7.59)	+0.51 (−7.94 to +8.95)
GzB+ Ki-67+ (% of GzB+)	2.55 (1.88–3.44)	5.44 (3.59–7.62)	3.84 (3.13–6.21)	4.44 (2.22–5.68)	4.83 (3.33–7.96)	4.30 (2.68–6.13)	+1.21 (−3.09 to +5.51)	+3.31 (−1.03 to +7.64)	+2.10 (−2.24 to +6.43)
GzB+ PD-1+ (% of GzB+)	10.75 (6.56–15.25)	8.52 (1.66–14.39)	5.52 (2.85–13.95)	9.12 (2.22–14.71)	7.57 (3.91–11.59)	10.35 (4.35–16.29)	−0.10 (−4.93 to +4.73)	−3.08 (−7.90 to +1.74)	−2.98 (−7.88 to +1.92)
IFNγ+ (% of CD8+)	0.55 (0.29–0.81)	0.71 (0.50–1.07)	0.72 (0.28–1.31)	0.65 (0.43–0.97)	0.52 (0.22–0.74)	0.39 (0.17–0.76)	+0.52 (−0.23 to +1.26)	**+1.16 (+0.39 to +1.92)**	+0.64 (−0.14 to +1.42)
Ki-67+ (% of CD8+)	3.44 (2.89–4.09)	7.00 (5.10–9.28)	5.51 (3.80–7.22)	6.58 (3.39–8.88)	7.24 (4.78–10.49)	6.71 (3.87–8.44)	+1.66 (−3.13 to +6.44)	+4.57 (−0.30 to +9.45)	+2.91 (−2.06 to +7.89)
PD-1+ (% of CD8+)	16.21 (9.54–18.88)	11.48 (3.62–17.91)	7.88 (4.03–16.90)	17.26 (8.84–25.04)	10.59 (6.80–15.34)	14.25 (7.33–20.36)	+1.98 (−4.25 to +8.21)	−1.22 (−7.59 to +5.16)	−3.20 (−9.64 to +3.24)
PD-1+ Ki-67+ (% of CD8+)	1.08 (0.72–1.50)	2.02 (0.94–2.95)	1.13 (0.64–1.74)	1.62 (0.90–2.81)	2.34 (0.99–3.57)	1.79 (1.22–3.17)	+0.53 (−2.05 to +3.12)	+1.89 (−0.74 to +4.53)	+1.36 (−1.27 to +3.99)

Values are medians (IQR). n—number of patients. Differences are adjusted for responder minus non-responder differences in change from the mixed-effects model. Positive estimates indicate a greater increase in responders. Bold values indicate *p* < 0.05. CI—confidence interval.

**Table 3:** Peripheral blood CD8+ T cell changes according to radiological response in the control group.

Population	Responders			Non-Responders			Difference in Change (95% CI)		
	Baseline (n = 7)	Week 3 (n = 7)	Week 6 (n = 7)	Baseline (n = 35)	Week 3 (n = 28)	Week 6 (n = 28)	Baseline to Week 3	Baseline to Week 6	Week 3 to Week 6
CD45RO+ (% of CD8+)	36.58 (32.17–47.25)	39.56 (34.22–47.78)	39.00 (25.47–49.98)	42.15 (24.05–52.09)	41.45 (30.93–58.10)	40.39 (32.00–52.97)	−1.02 (−14.28 to +12.24)	−0.45 (−13.71 to +12.81)	+0.57 (−12.75 to +13.89)
CD28+ (% of CD8+)	30.00 (24.23–53.56)	45.00 (36.00–53.41)	32.10 (28.69–48.92)	43.90 (25.91–54.61)	36.38 (26.98–51.34)	36.00 (21.74–49.70)	−4.05 (−23.28 to +15.19)	+3.85 (−12.10 to +19.80)	+7.90 (−11.10 to +26.89)
CD28+ Ki-67+ (% of CD28+)	2.35 (1.40–2.83)	3.23 (1.86–6.65)	4.04 (2.34–4.80)	2.29 (1.02–3.20)	2.72 (1.15–4.92)	1.60 (1.14–3.56)	+2.01 (−0.27 to +4.28)	+1.79 (−0.48 to +4.07)	−0.21 (−2.49 to +2.07)
CD28+ PD-1+ (% of CD28+)	4.88 (2.45–8.18)	5.54 (2.66–11.66)	1.97 (1.14–9.17)	4.68 (3.64–7.92)	5.06 (2.99–6.55)	3.88 (2.24–6.45)	−0.14 (−3.75 to +3.47)	−1.07 (−4.51 to +2.38)	−0.93 (−4.54 to +2.68)
GzB+ (% of CD8+)	66.59 (49.70–78.14)	64.49 (50.92–80.39)	58.44 (52.23–67.97)	65.42 (40.16–77.50)	62.63 (47.11–79.87)	61.67 (39.97–81.37)	−1.21 (−8.85 to +6.43)	−2.55 (−10.19 to +5.09)	−1.34 (−8.98 to +6.31)
GzB+ Ki-67+ (% of GzB+)	2.42 (1.61–3.32)	4.97 (3.69–10.09)	3.95 (2.31–6.88)	3.08 (1.83–3.77)	5.54 (3.09–7.93)	3.81 (2.96–5.57)	+2.16 (−1.89 to +6.21)	+1.10 (−3.12 to +5.32)	−1.06 (−5.29 to +3.17)
GzB+ PD-1+ (% of GzB+)	10.34 (7.93–14.46)	10.80 (9.05–14.47)	6.54 (4.04–12.80)	6.89 (4.64–10.58)	7.13 (5.46–11.35)	5.88 (3.79–9.16)	−1.81 (−6.53 to +2.90)	−2.64 (−7.35 to +2.08)	−0.82 (−5.55 to +3.90)
IFNγ+ (% of CD8+)	0.52 (0.23–0.58)	0.83 (0.69–0.91)	0.65 (0.34–0.76)	0.39 (0.22–0.87)	0.60 (0.25–0.89)	0.65 (0.27–1.20)	+0.19 (−0.36 to +0.73)	−0.23 (−0.78 to +0.31)	−0.42 (−0.97 to +0.13)
Ki-67+ (% of CD8+)	3.95 (3.34–4.70)	7.89 (4.59–17.34)	6.01 (5.00–8.73)	4.03 (2.90–5.12)	8.02 (4.99–10.93)	5.65 (4.21–8.50)	+2.80 (−2.43 to +8.03)	+4.16 (−1.07 to +9.39)	+1.36 (−3.90 to +6.63)
PD-1+ (% of CD8+)	12.14 (10.70–24.71)	12.64 (11.88–19.10)	8.10 (4.62–19.10)	10.46 (7.71–20.22)	9.89 (7.79–16.18)	8.69 (5.54–12.19)	−2.22 (−8.45 to +4.00)	−2.75 (−8.98 to +3.47)	−0.53 (−6.78 to +5.72)
PD-1+ Ki-67+ (% of CD8+)	1.28 (0.98–1.40)	2.17 (1.28–5.76)	1.19 (0.79–1.77)	1.06 (0.65–1.92)	1.78 (1.34–3.30)	1.21 (0.81–2.33)	+1.31 (−0.39 to +3.02)	+0.57 (−1.14 to +2.28)	−0.74 (−2.45 to +0.96)

Values are medians (IQR). n—number of patients. Differences are adjusted for responder minus non-responder differences in change from the mixed-effects model. Positive estimates indicate a greater increase in responders. CI—confidence interval.

**Table 4:** Univariate and multivariable Cox analyses of progression-free survival and overall survival.

T Cell Changes at Week 3	Univariate PFS *p*-Value	Multivariable PFS HR (95% CI)	Multivariable PFS *p*-Value	PFS PH *p*-Value	Univariate OS *p*-Value	Multivariable OS HR (95% CI)	Multivariable OS *p*-Value	OS PH *p*-Value
CD45RO+	0.618	0.77 (0.49–1.21)	0.258	0.603	0.409	0.66 (0.36–1.19)	0.167	0.699
CD28+	0.737	1.20 (0.72–1.98)	0.485	0.796	0.136	1.72 (0.91–3.27)	0.095	0.310
CD28+Ki-67+	0.197	0.87 (0.66–1.15)	0.341	0.637	0.506	0.94 (0.68–1.29)	0.685	0.653
CD28+PD-1+	0.346	0.82 (0.61–1.11)	0.196	0.921	0.533	1.05 (0.73–1.49)	0.806	0.258
GzB+	0.943	0.92 (0.28–3.01)	0.893	0.782	0.609	0.73 (0.18–2.96)	0.660	0.467
GzB+Ki-67+	0.012	0.72 (0.54–0.96)	0.024	0.425	0.023	0.72 (0.52–0.99)	0.046	0.783
GzB+PD-1+	0.692	1.01 (0.78–1.30)	0.960	0.038	0.170	1.22 (0.87–1.71)	0.256	0.215
IFNγ+	0.209	0.90 (0.71–1.15)	0.416	0.825	0.478	0.96 (0.72–1.27)	0.750	0.682
Ki-67+	0.072	0.76 (0.55–1.04)	0.090	0.641	0.208	0.79 (0.55–1.13)	0.190	0.914
PD-1+	0.894	0.92 (0.67–1.27)	0.602	0.290	0.402	1.09 (0.72–1.65)	0.667	0.323
PD-1+Ki-67+	0.030	0.78 (0.61–0.99)	0.039	0.151	0.373	0.89 (0.67–1.17)	0.394	0.359

Week 3 analyses used a 21-day landmark. HRs represent the association per doubling of the week 3 to baseline ratio. PFS—Progression-free survival; OS—overall survival; HR—Hazard ratio; 95% CI—95% confidence interval; PH—proportional hazards.

## Data Availability

Data necessary for statistical analysis and duplication are provided in the manuscript and supplementary files. Any additional data presented in this study are available on request from the corresponding author.

## Supplementary Materials

The following supporting information can be downloaded at: https://www.mdpi.com/article/10.3390/cancers18111793/s1, Table S1: Observed values and within-group changes for CD8+ T cell subsets by treatment group; Table S2: Differences in CD8+ T cell subset dynamics by treatment group; Table S3: Landmark analysis population details; Table S4: Univariate and multivariable Cox analyses of progression-free survival and overall survival.

## Abbreviations

The following abbreviations are used in this manuscript:

CI	Confidence interval.
HR	Hazard ratio
GzB	Granzyme B
ICI	Immune checkpoint inhibitor
IFNγ	Interferon-γ
IQR	Interquartile range
NSCLC	Non-small cell lung cancer
OS	Overall survival
PD	Progressive disease
PD-1	Programmed cell death protein 1
PD-L1	Programmed death-ligand 1
PFS	Progression-free survival
PH	Proportional Hazards
PR	Partial response
RECIST	Response Evaluation Criteria In Solid Tumors
SD	Stable disease
TPS	Tumor proportion score
