# Supplementary material for: Peripheral CD8+ T Cell Dynamics and Clinical Outcomes in Metastatic Non-Small Cell Lung Cancer Following Bronchoscopic Cryotherapy and Pembrolizumab-Based Therapy
Source: Cancers (Basel). 2026 May 31;18(11):1793. doi: 10.3390/cancers18111793 (PMC13255720; doi:10.3390/cancers18111793)
Supplement: Supplementary file 1 [file cancers-18-01793-s001.zip › cancers-4275742-supplementary.pdf]

Supplemental Table S1. Observed values and within-group changes for CD8+ T cell subsets by treatment group.

					Change (95% CI), p-value		
**Population**	**Group**	**Baseline**	**Week 3**	**Week 6**	**Baseline to week 3**	**Baseline to week 6**	**Week 3 to week 6**
CD45RO+	Cryotherapy	42.48 (34.41–50.21)	45.17 (30.13–51.50)	41.31 (33.35–49.88)	+0.27 (-4.86 to +5.40); p=0.918	+0.09 (-5.16 to +5.35); p=0.973	-0.18 (-5.50 to +5.14); p=0.948
CD45RO+	Control	41.58 (25.33–51.50)	40.90 (31.37–53.89)	39.00 (31.00–53.09)	+4.14 (-0.58 to +8.86); p=0.085	+1.88 (-2.84 to +6.60); p=0.434	-2.26 (-7.07 to +2.55); p=0.357
CD28+	Cryotherapy	38.73 (24.40–50.46)	37.39 (21.44–49.95)	30.44 (21.67–42.43)	-4.40 (-9.98 to +1.17); p=0.122	**-9.70 (-15.14 to -4.26); p=<0.001**	-5.29 (-10.85 to +0.26); p=0.062
CD28+	Control	41.00 (25.65–54.67)	37.30 (27.87–52.23)	35.00 (23.18–51.40)	-3.29 (-9.07 to +2.48); p=0.264	-2.47 (-7.95 to +3.01); p=0.376	+0.82 (-4.92 to +6.56); p=0.779
CD28+Ki-67+	Cryotherapy	1.85 (1.31–3.43)	3.07 (1.71–6.58)	2.67 (1.31–4.93)	+1.07 (-0.07 to +2.21); p=0.066	+0.67 (-0.49 to +1.84); p=0.258	-0.40 (-1.58 to +0.79); p=0.512
CD28+Ki-67+	Control	2.29 (1.05–3.22)	2.80 (1.32–5.14)	2.17 (1.29–3.95)	**+1.40 (+0.35 to +2.45); p=0.009**	+0.70 (-0.35 to +1.75); p=0.190	-0.70 (-1.77 to +0.37); p=0.201
CD28+PD-1+	Cryotherapy	6.22 (4.00–8.90)	5.81 (1.62–7.24)	4.29 (2.05–6.43)	-1.43 (-2.89 to +0.03); p=0.056	**-1.99 (-3.48 to -0.51); p=0.008**	-0.56 (-2.06 to +0.94); p=0.461
CD28+PD-1+	Control	4.73 (3.11–7.92)	5.11 (2.96–7.76)	3.86 (1.78–7.36)	-0.22 (-1.57 to +1.14); p=0.751	**-1.41 (-2.75 to -0.07); p=0.040**	-1.19 (-2.54 to +0.17); p=0.085
GzB+	Cryotherapy	66.91 (49.89–72.00)	67.81 (53.34–75.44)	72.52 (61.01–80.09)	+2.52 (-1.10 to +6.13); p=0.172	**+5.39 (+1.68 to +9.10); p=0.004**	+2.87 (-0.85 to +6.60); p=0.131
GzB+	Control	65.59 (41.80–77.75)	64.49 (46.69–79.91)	58.44 (41.80–80.50)	+2.22 (-1.12 to +5.56); p=0.193	+0.06 (-3.28 to +3.40); p=0.973	-2.16 (-5.52 to +1.20); p=0.208
GzB+Ki-67+	Cryotherapy	3.24 (1.92–4.99)	4.83 (3.42–7.65)	4.09 (2.78–6.44)	**+2.14 (+0.18 to +4.10); p=0.032**	**+2.53 (+0.55 to +4.52); p=0.012**	+0.39 (-1.59 to +2.37); p=0.698
GzB+Ki-67+	Control	3.06 (1.75–3.67)	5.41 (3.42–7.96)	3.85 (2.61–5.74)	**+3.14 (+1.34 to +4.94); p=<0.001**	+0.92 (-0.91 to +2.75); p=0.325	**-2.22 (-4.07 to -0.37); p=0.018**
GzB+PD-1+	Cryotherapy	10.23 (5.37–14.98)	8.52 (2.42–11.86)	7.39 (3.35–14.45)	-1.66 (-3.89 to +0.57); p=0.145	-1.05 (-3.28 to +1.18); p=0.356	+0.61 (-1.65 to +2.87); p=0.598
GzB+PD-1+	Control	7.65 (5.56–11.03)	8.58 (5.47–13.43)	6.11 (3.75–9.70)	-0.68 (-2.71 to +1.36); p=0.514	**-2.41 (-4.44 to -0.37); p=0.020**	-1.73 (-3.78 to +0.32); p=0.098
IFNγ+	Cryotherapy	0.57 (0.40–0.91)	0.57 (0.31–0.86)	0.55 (0.24–0.97)	-0.09 (-0.41 to +0.22); p=0.557	+0.17 (-0.15 to +0.49); p=0.302	+0.26 (-0.07 to +0.59); p=0.116
IFNγ+	Control	0.39 (0.22–0.78)	0.66 (0.31–0.89)	0.65 (0.26–1.07)	+0.19 (-0.10 to +0.48); p=0.191	+0.28 (-0.01 to +0.57); p=0.056	+0.09 (-0.21 to +0.39); p=0.562
PD-1+	Cryotherapy	16.37 (8.46–22.00)	10.79 (5.40–16.03)	10.16 (4.84–17.84)	**-4.80 (-7.64 to -1.96); p=<0.001**	**-4.01 (-6.92 to -1.10); p=0.007**	+0.80 (-2.15 to +3.74); p=0.597
PD-1+	Control	11.08 (7.97–20.33)	11.30 (8.15–16.85)	8.50 (5.32–13.18)	-1.41 (-4.03 to +1.20); p=0.289	**-4.11 (-6.73 to -1.50); p=0.002**	**-2.70 (-5.37 to -0.03); p=0.047**
Ki-67+	Cryotherapy	4.08 (2.89–7.65)	7.24 (5.01–9.72)	5.65 (3.84–7.92)	**+2.35 (+0.02 to +4.67); p=0.048**	**+2.90 (+0.52 to +5.27); p=0.017**	+0.55 (-1.87 to +2.98); p=0.656
Ki-67+	Control	4.03 (3.13–5.08)	7.94 (4.87–11.36)	5.90 (4.24–8.62)	**+4.39 (+2.26 to +6.52); p=<0.001**	**+2.49 (+0.36 to +4.62); p=0.022**	-1.90 (-4.10 to +0.29); p=0.090
PD-1+Ki-67+	Cryotherapy	1.21 (0.81–1.86)	2.17 (0.89–3.19)	1.36 (0.69–2.48)	+0.68 (-0.34 to +1.71); p=0.192	**+1.18 (+0.13 to +2.23); p=0.027†**	+0.50 (-0.55 to +1.55); p=0.352
PD-1+Ki-67+	Control	1.13 (0.70–1.79)	1.79 (1.33–3.38)	1.20 (0.81–2.16)	**+1.56 (+0.60 to +2.51); p=0.001**	+0.49 (-0.46 to +1.44); p=0.310	**-1.06 (-2.01 to -0.11); p=0.028**

Values are medians (IQR). Differences are adjusted percentage point changes estimated from mixed-effects model. Positive estimates indicate a greater increase from previous time-point. Bold values indicate p<0.05. † indicates a statistically significant original contrast that was not retained in the log-transformed sensitivity analysis. CI – confidence interval.

Supplemental Table S2. Differences in CD8+ T cell subset dynamics by treatment group.

Population	Difference in change (95% CI), p-value		
	Baseline to week 3	Baseline to week 6	Week 3 to week 6
CD45RO+	-3.87 (-10.84 to +3.10); p=0.276	-1.79 (-8.85 to +5.27); p=0.619	+2.08 (-5.09 to +9.26); p=0.569
CD28+	-1.11 (-9.14 to +6.92); p=0.787	-7.22 (-14.94 to +0.50); p=0.067	-6.11 (-14.10 to +1.87); p=0.133
CD28+Ki-67+	-0.33 (-1.88 to +1.22); p=0.678	-0.03 (-1.60 to +1.54); p=0.973	+0.30 (-1.29 to +1.90); p=0.711
CD28+PD-1+	-1.21 (-3.21 to +0.78); p=0.234	-0.59 (-2.59 to +1.41); p=0.564	+0.62 (-1.40 to +2.64); p=0.545
GzB+	+0.30 (-4.62 to +5.22); p=0.905	**+5.33 (+0.34 to +10.32); p=0.036**	**+5.03 (+0.01 to +10.05); p=0.049**
GzB+Ki-67+	-1.00 (-3.66 to +1.66); p=0.462	+1.61 (-1.08 to +4.31); p=0.241	+2.61 (-0.10 to +5.32); p=0.059
GzB+PD-1+	-0.98 (-4.00 to +2.03); p=0.523	+1.36 (-1.66 to +4.37); p=0.378	+2.34 (-0.72 to +5.39); p=0.133
IFNγ+	-0.29 (-0.71 to +0.14); p=0.188	-0.11 (-0.54 to +0.32); p=0.614	+0.18 (-0.27 to +0.62); p=0.439
PD-1+	-3.39 (-7.25 to +0.47); p=0.085	+0.11 (-3.81 to +4.02); p=0.958	+3.49 (-0.48 to +7.47); p=0.085
Ki-67+	-2.05 (-5.20 to +1.10); p=0.203	+0.41 (-2.78 to +3.60); p=0.802	+2.46 (-0.82 to +5.73); p=0.141
PD-1+Ki-67+	-0.87 (-2.27 to +0.53); p=0.222	+0.69 (-0.73 to +2.11); p=0.341	**+1.56 (+0.14 to +2.98); p=0.031†**

Values are model-derived differences from mixed-effects model. Positive estimates indicate a greater increase, or smaller decrease in the cryotherapy group compared with controls. Bold values indicate p<0.05. † indicates a statistically significant original contrast that was not retained in the log-transformed sensitivity analysis. CI – confidence interval.

Supplemental Table S3. Landmark analysis population details.

Landmark	Outcome	Patients included	Events after landmark	Excluded due to missing paired samples	Fitted parameters	EPV
Week 3	PFS	65	50	11	4	12.50
	OS	65	37	11	4	9.25
Week 6	PHS	63	48	13	4	12.00
	OS	63	35	13	4	8.75

PFS—Progression-free survival; OS—overall survival; EPV—events per variable.

Supplemental Table S4. Univariate and multivariable Cox analyses of progression-free survival and overall survival.

T cell changes at Week 3	Univariate PFS p-value	Multivariable PFS HR (95% CI)	Multivariable PFS p-value	PFS PH p-value	Univariate OS p-value	Multivariable OS HR (95% CI)	Multivariable OS p-value	OS PH p-value
CD45RO+	0.240	0.64 (0.40–1.02)	0.060	0.937	0.177	0.54 (0.29–1.01)	0.053	0.586
CD28+	0.835	0.96 (0.55–1.66)	0.882	0.878	0.366	1.27 (0.67–2.40)	0.458	0.037
CD28+Ki-67+	0.098	0.87 (0.69–1.10)	0.255	0.229	0.429	0.95 (0.69–1.30)	0.749	0.490
CD28+PD-1+	0.626	0.84 (0.64–1.11)	0.224	0.006	0.622	0.99 (0.72–1.37)	0.968	0.863
GzB+	0.636	1.71 (0.60–4.92)	0.316	0.049	0.679	1.79 (0.59–5.45)	0.304	0.056
GzB+Ki-67+	0.445	0.97 (0.72–1.29)	0.819	0.228	0.427	0.94 (0.64–1.37)	0.751	0.759
GzB+PD-1+	0.699	1.01 (0.79–1.27)	0.963	0.343	0.334	1.09 (0.79–1.49)	0.609	0.914
IFNγ+	0.902	1.07 (0.85–1.34)	0.591	0.745	0.235	1.27 (0.96–1.69)	0.093	0.500
Ki-67+	0.566	0.97 (0.71–1.31)	0.827	0.098	0.702	0.95 (0.64–1.41)	0.815	0.592
PD-1+	0.955	0.94 (0.71–1.26)	0.690	0.021	0.710	0.95 (0.65–1.40)	0.812	0.705
PD-1+Ki-67+	0.190	0.86 (0.69–1.06)	0.159	0.785	0.456	0.87 (0.66–1.16)	0.344	0.955

Week 6 analyses used a 42-day landmark. HRs represent the association per doubling of the week 6 to baseline ratio. PFS—Progression-free survival; OS—overall survival; HR —Hazard ratio; 95% CI—95% confidence interval; PH—proportional hazards.
